# HER2/neu and Ki-67 as prognostic biomarkers in urothelial carcinoma

**DOI:** 10.6026/973206300220885

**Published:** 2026-02-28

**Authors:** Anamika Rawat, Himanshu Joshi, Nishant Mitra, Rashmi Bhardwaj

**Affiliations:** 1 Department of Pathology, Kalyan Singh Government Medical College, Bulandshahr, Uttar Pradesh, India; 2 Department of Pathology, Uttar Pradesh University of Medical Sciences (U.P.U.M.S.), Saifai, Etawah, Uttar Pradesh, India; 3 Department of Pathology, ESIC Model Hospital, Basai Darapur, Delhi, India; 4 Department of Anatomy, Shri Siddhi Vinayak Medical College and Hospital, Sambhal, Uttar Pradesh, India

**Keywords:** Immunohistochemistry (IHC), trans-urethral resection of bladder tissue (TURBT), urothelial carcinoma

## Abstract

The prognostic significance of Her2/neu and Ki-67 expression remains insufficiently defined in urothelial carcinoma even though it constitutes a major proportion of malignancies in the male population of the United States and India. Therefore, it is of interest to evaluate Immunohistochemical (IHC) expression of HER2/neu and Ki-67 to correlate with clinicopathological parameters in thirty histologically confirmed cases of urothelial carcinoma in an Indian cohort. We show that the overexpression of HER2/neu and Ki-67 correlates positively with higher tumor grade, advanced stage and may serve as reliable prognostic biomarkers thus guiding therapeutic decision making in management of urothelial carcinoma.

## Background:

Urothelial carcinoma ranks among the most common genitourinary malignancies among men in the United States and India, with rising incidence linked to environmental and occupational risk factors such as smoking and industrial exposures [1, 2]. Although HER2/neu overexpression has been shown to be associated with poor prognosis and is a proposed therapeutic target, variability in Western and East Asian studies and limited Indian data necessitate further evaluation of these biomarkers hence the rationale of this study [3, 4]. Therefore, it is of interest to describe HER2/neu and Ki-67 as Prognostic Biomarkers in Urothelial Carcinoma.

## Materials and Methods:

This cross-sectional, analytical study was carried out at a tertiary care hospital center over a three-year period after approval by the Institutional Ethics Committee (Ref. No. IEC/053). Thirty histologically confirmed urothelial carcinoma cases were selected after informed consent.

## Routine histopathology:

Routine histopathology was carried out using paraffin embedded tissue sectioning and H & E staining. The interpretation was done using the latest edition of the tumor, node and sand and metastasis (TNM) classification system developed by American joint committee on cancer (AJCC).

## Immunohistochemistry:

For IHC, polyclonal rabbit anti-human HER2/neu and Ki-67 Oncoprotein (Agilent technologies, Inc., Santa Clara, USA) were utilized, with heat-induced epitope retrieval by microwave processing for fifteen minutes and counterstaining with diluted Harris hematoxylin (Millipore Sigma, St. Louis, USA). Interpretation of HER2/neu staining was done in accordance with the American Society of Clinical Oncology/College of American Pathologists (ASCO/CAP) criteria for breast carcinoma. IHC scoring was double-blinded and for IHC interpretation of HER2/neu and Ki-67, depending on staining intensity and number of cells stained, grading from 0 to 3 was used.

## Inclusion criteria:

Urinary bladder biopsies reported malignancies on histopathology were included in the study.

## Exclusion criteria:

Samples with unavailable clinicopathological data, autolysed specimen and bladder biopsies found inadequate and bladder biopsies with inflammatory/metastatic lesions were excluded from our study.

## Statistical analysis:

This minimum of thirty cases was included so as to achieve preliminary statistical power of 80%. Data collected was subjected to statistical analysis using chi-square test and analysis of variance (ANOVA) test. Statistical product and service solutions (SPSS) software version 24 was used for analysis. Correlation between IHC staining and various clinical parameters such as grade, stage and histological type were sought and if found, P values were calculated for establishing the correlation. P value < 0.05 was considered statistically significant.

## Results and Discussion:

In the present study, of the total thirty samples, male subjects were twenty-five (83.3%) and five were females (16.67%) giving a male: female ratio of 5:1. The age of the patients ranged from 46 to 78 years. Of the significant epidemiological factors, smoking history was present in twenty-one subjects (70%). As per histology, the most common histological type of cancer was the transitional cell carcinoma seen in twenty-seven subjects (90%), while only two cases (6.67%) were of squamous cell carcinoma histology and only one sample (3.33%) was of sarcomatoid histology. Among the subjects, as per AJCC 8th edition twenty-two (71.33%) patients were in stage 1, followed by seven subjects (23.33%) in stage 2 and only one patient (3.33%) was of stage 3. As per WHOM grading, twenty patients (66.67%) had high grade urothelial carcinoma (HGUC) and ten patients (33.33%) had low grade urothelial carcinoma (LGUC). The expression of HER2/ neu and Ki-67 is summarized in Table 1 and Table 2 respectively. Table 1 shows HER2/neu expression correlating significantly with tumour type (p = .008), stage (p = .005) and grade (p = 0.007). Table 2 shows Ki-67 expression correlating significantly with tumour type (p = .005), stage (p < .01) and grade (p = 0.002). Overall, 33.3% of cases demonstrated overexpression of HER2/neu and Ki-67. Both markers showed a strong positive correlation (r = 0.71, p < 0.001) and an emerging therapeutic implication [5]. These findings are in alignment with previous studies thus indicating that HER2/neu overexpression and increased proliferation indices are linked to worse clinical outcomes in urothelial carcinoma [6, 7]. Recent studies by Raggi *et al.* (2026), Coca Membribes *et al.* (2026), Nadal *et al.* (2024) and Ehrlich *et al.* (2025) highlights the expanding role of biomarker-driven therapies and the prognostically significant role of molecular profiling in management of urothelial carcinoma [8, 9, 10-11].

## Conclusion:

HER2/neu and Ki-67 overexpression significantly correlate with tumor type, grade and stage thus suggesting their potential role as prognostic markers in urothelial carcinoma. Their combined evaluation may identify high-risk patients suitable for targeted anti HER2/neu therapies or intensified surveillance.

## Advancement to knowledge:

In a limited cohort of urothelial carcinoma, this study provides preliminary evidence of the correlation of Her2/neu and Ki-67 expression with tumor grade and stage, reinforcing their potential role as adjunct prognostic biomarkers.

## Limitations:

Single institutional study on a small sample size limits generalizability; HER2/neu gene amplification studies via fluorescence in-situ hybridization (FISH) could further validate IHC findings.

## Future scope:

Large scale multicentre studies are needed to validate HER2/neu and Ki-67 as combined prognostic markers and to explore their role in selecting candidates for targeted therapy. Ki-67 and HER/2neu co-expression, may be superior to single marker expression in predicting tumor prognosis.

## Figures and Tables

**Table 1:** Association of her2/neu expression with pathological variables

**Variables**	**Number (N)**	**HER-2 Expression Status**								**P value**
		**0**		**Grade 1**		**Grade 2**		**Grade 3**		
		**N**	**Percentage (%)**	**N**	**%**	**N**	**%**	**N**	**%**	
**Type**										
TCC	27	2	7.4	9	33.3	7	25.9	9	33.3	
Sarcomatoid UC	1	0	0	0	0	0	0	1	100	0.008*
SCC	2	2	100	0	0	0	0	0	0	
**Grade**										
High Grade	20	0	0	5	25	6	30	9	45	0.007*
Low Grade	10	4	40	4	40	1	10	1	10	
**Stage**										
Stage 1	22	4	18.2	8	36.4	5	22.7	5	22.7	0.005*
Stage 2	7	0	6.7	1	13.3	2	26.7	4	53.3	
Stage 3	1	0	0	0	0	0	0	1	100	
Total	30	4	13.3	9	30	7	23.3	10	33.3	
*statistically
significant

**Table 2:** Association of ki-67 expression with pathological variables

**Variables**	**N**	**Ki-67 Expression Status**								**P value**
		**0**		**Grade 1**		**Grade 2**		**Grade 3**		
		**N=3**	**%**	**N=8**	**%**	**N=9**	**%**	**N=10**	**%**	
**Type**										
TCC	27	1	3.7	8	29.6	9	33.3	9	33.3	
Sarcomatoid UC	1	0	0	0	0	0	0	1	100	0.005*
SCC	2	2	100	0	0	0	0	0	0	
**Grade**										
High Grade	20	0	0	3	15	7	35	10	50	0.002*
Low Grade	10	3	30	5	50	2	20	0	0	
**Stage**										
Stage 1	22	3	13.6	7	31.8	8	36.4	4	18.2	<0.01*
Stage 2	7	0	0	1	14.3	1	14.3	5	71.4	
Stage 3	1	0	0	0	0	0	0	1	100	
Total	30	3	10	8	26.7	9	30	10	33.3	
*statistically
significant
